# Effect of Social Factors and the Natural Environment on the Etiology and Pathogenesis of Diabetes Mellitus

**DOI:** 10.1155/2019/8749291

**Published:** 2019-06-25

**Authors:** Guangtong Dong, Lianlian Qu, Xuefeng Gong, Bing Pang, Weitian Yan, Junping Wei

**Affiliations:** ^1^Guang'anmen Hospital, China Academy of Traditional Chinese Medicine, Beijing, China; ^2^Penglai Traditional Chinese Hospital, Shandong, China; ^3^The Third Affiliated Hospital of Beijing University of Chinese Medicine, Beijing, China

## Abstract

Type 2 diabetes mellitus (T2DM) is currently a public health problem worldwide and a threat to human health and social development. The incidence rate of the disease is steadily increasing. Various genetic and environmental factors have been established as influencing the pathogenesis of this disease. However, the influence of social factors and the natural environment on DM incidence should also be considered. Low-grade inflammation could represent a central point of connection integrating all these potential triggers, being partly responsible for the development of insulin resistance. This paper aims to elaborate on the impact of the natural environment and social factors on DM development, with a special focus on six aspects of the pathogenesis of DM: pollution, radiation, psychology, drink, sleep, and exercise. We identified a two-way relationship between T2DM and social and natural environments. Changes in these environments may lead to low-grade inflammation, which in turn induces or aggravates T2DM and vice versa. Poor lifestyle may lead to increased insulin resistance and promote DM development. Improvements in blood glucose control can be achieved through nonenvironmental and behavioral interventions.

## 1. Introduction

Diabetes mellitus (DM) is currently the third most dangerous chronic noncommunicable disease in terms of its effect on human health [1]. Worldwide, the prevalence of the disease is continuously increasing, owing in part to rapid social economy developments, improved living standards, acceleration of urbanization, industrialization, and increase in the proportion of aging individuals. According to the World Health Organization, about 422 million people are currently living with diabetes; this number is expected to increase to 600 million by 2040 [2]. The prevalence rate of DM among adults in China is 11.6% [3]. The dramatic increase in the number of people with DM is among the greatest challenges facing the healthcare sector today. The etiology and pathogenesis of DM are extremely complicated. At present, genetic factors, environmental factors (obesity), and interaction of these two factors have been established as increasing the risk of type 2 diabetes mellitus (T2DM) [4]. However, various social factors and the natural environment may also affect the synthesis and secretion of insulin. Some scholars have discovered that, in a similar setting, low-grade inflammation could represent a central point of connection integrating all these potential triggers, being partly responsible for the development of insulin resistance in recent studies, and may represent a sensor of the consequence of metabolic imbalance [5, 6]. T2DM is associated with an increased inflammatory score. The term ‘metaflammation' was coined to indicate metabolically triggered inflammation [7]. These six factors are mainly related to T2DM. This paper mainly aims to demonstrate the impact of external and internal factors on the development of T2DM from the perspective of the influence of the natural environment and social factors on individual behavior.

## 2. Air Pollution

Air pollution is becoming an increasingly serious problem worldwide. Air pollutants can be classified as gaseous pollutants (sulfur compounds, nitrogen compounds, carbon oxides, etc.) and atmospheric particulate matter (PM_10_ and PM_2.5_). Several studies have found that environmental factors play an important role in the pathogenesis of T2DM; specifically, air pollution has been shown to be an important catalyst [8–10]. Andersen et al. [11], in their 9.7-month follow-up study of 51,818 volunteers, demonstrated that people with healthy lifestyles and who regularly exercised had a lower incidence of T2DM than those without these habits in a population of individuals with long-term exposure to traffic-related pollutants. Traffic-related pollutants are among the potential risk factors for T2DM development. Dijkema et al. [12] conducted a cross-sectional study in which 8,018 elderly people were screened for DM. They concluded that the odds of developing type 2 DM increased to 1.39 when NO_2_ level in the atmosphere increased to 10 mg·m^−3^. A Chinese study [13] with a large sample showed that the type 2 DM risk index was 1.11 (95% confidence interval [CI]: 1.02–1.21) when the PM_2.5_ level increased to an average concentration of 10 *μ*g·m^−3^. Evidence-based medicine supports the idea that air pollution is a risk factor for T2DM, while epidemiological evidence indicates that greater increases in the level of air pollution can cause or increase the prevalence of T2DM. The pathogenesis associated with this increase in the prevalence of T2DM may be attributed to stress-mediated insulin resistance and/or decreased insulin sensitivity, which results in T2DM development through inflammatory response, oxidative stress, and endoplasmic reticulum. The presence of air pollutants in the lungs can stimulate alveolar epithelial cells and macrophages to produce inflammatory factors, such as interleukin and macrophage inflammatory protein 2, and lead to the disorderly interaction of other mediators of systemic inflammatory response [14]. During the process, islet inflammation leads to the destruction or apoptosis of islet cells and insulin sensitivity is reduced, which affects the utilization of glucose by peripheral tissues, thereby increasing the risk of DM. At the same time, when the lungs are stimulated by external pollutants (such as PM_2.5_), they release reactive oxygen species (ROS) rapidly; the overaccumulation of ROS to levels that exceed those that can be normally removed by the body disrupts the balance between the oxidation and antioxidant systems, resulting in oxidative damage to the tissues and organs [15]. Islet *β*-cells, which are sensitive to ROS, have low levels of antioxidant enzymes and poor antioxidant capacity. As a result, ROS can directly damage *β*-cells and lead to DM. Studies have shown that PM_10_ can increase the proportion of immune cells, malondialdehyde, and neutrophil chemotactic factors in the bronchoalveolar lavage fluid in rats, proving that it can enhance inflammation and the role of oxidative stress in rodents [16]. Islet *β*-cells are rich in endoplasmic reticulum, and air pollution can mediate endoplasmic reticulum stress. If the stress persists for long periods, the cells initiate an unfolded protein response, activate the apoptotic pathway, induce islet *β*-cell apoptosis, and affect insulin secretion, leading to T2DM.

## 3. Radiation Environment

Mobile phones, computers, electronic watches, and other communication equipment have become indispensable in daily life. Radiotherapy is an important treatment modality for cancer. The development of the military industry and demand for alternative energy sources have also led to the establishment of a large number of nuclear power plants. However, long-term exposure to cell phone towers, smart meters, and other radiation-emitting devices can adversely affect human health. Most people know little about the possible relationship between radiation exposure and diabetes [17]. For example, among 8 articles including a systematic review and meta-analysis of 1863 T2DM patients reported by Pettit et al. which identified and analyzed the current evidence on glycemic control (HbA1c) during and after cancer treatment, the effect of radiation on glycemic control is not mentioned [18]. However, it has been considered by the International Commission for Radiation Protection as one factor involved in multifactorial diseases [19].

Existing studies have shown that cancer patients receiving radiotherapy have an increased risk of developing insulin resistance and T2DM [20]. Patients undergoing radiotherapy in whom a dose to the pancreatic tail is 10 Gy or higher have a 11.5 times higher risk of developing DM than those without radiation therapy, suggesting that ionizing radiation exposure may lead to T2DM [20]. In China, a survey has shown that the incidence rates of obesity and metabolic syndrome among those working in nuclear power plants are significantly higher than the national average, indicating that such workers, even after retirement, may have a higher risk of DM than the general population in the corresponding age group [21]. Radiation is a major inducer of inflammatory responses [22]. While radical therapy is over within a short time of radiation exposure, the ensuing inflammatory response perpetuates the response by generating recurring waves of ROS, cytokines, chemokines, and growth factors with associated inflammatory infiltrates [23]. Systemic low-grade inflammatory response and a large number of inflammatory mediators are known pathological features of diabetes [7]. Overexpression of inflammatory factors such as serum C-reactive protein, interleukin (IL)-6, and tumor necrosis factor-*α* can inhibit insulin secretion and islet function and leads to insulin secretion disorder and insulin resistance, resulting in insufficient insulin secretion and inability to lower blood glucose levels, leading to T2DM and metabolic syndrome [24]. Therefore, we consider that inflammatory responses may be the putative mechanism linking radiation and T2DM.

While radiation exposure increases the risk of T2DM development, T2DM, in turn, increases the degree of radiation damage among cancer patients undergoing radiation therapy. Due to the presence of autonomic neuropathy in diabetic patients, the ability of the parasympathetic nerves to regulate bronchial activity is reduced, and the permeability of patients' blood vessel walls and sensitivity to ionizing radiation are enhanced, which is not conducive to the absorption and dissipation of inflammatory reactions. Ma et al. [25] showed that DM can aggravate, to an extent, the symptoms of radiation pneumonitis in lung cancer patients. Few studies have focused on the incidence of radiation-induced DM. However, through our review of the aforementioned studies, it can be concluded that radiation can cause diabetes. Diabetes onset may further aggravate the damage caused by radiation, forming a vicious circle. Radiation damage can stimulate the pathogenesis of diabetes; the mutual influence of the two requires further exploration.

## 4. Psychological Factors

The influence of psychological factors such as depression and anxiety on DM is increasingly being investigated, owing to gradual changes in the social medical model and increasing pressure of life. The “biological-psychological-social medical model" has led to the realization that social and psychological factors play a vital role in the process of DM development and the associated outcomes. According to a report published by the World Health Organization Diabetes Expert Committee, work-related and other psychological burdens may aggravate psychological and social pressures. Furthermore, this situation may induce and produce glucose tolerance-related abnormalities through hormonal action on insulin secretion and glucose metabolism [26]. Engum et al. showed that an increase in the incidence of type 2 DM in people with depressive symptoms regardless of sex in a follow-up survey of 37,291 people conducted over 10 years. Another conclusion is that depression is a significant risk factor for DM development [27]. A meta-analysis of 20 articles, including 45,514 people, showed that the risk of DM in people with stress was 1.80 times higher than that in the normal population [28], showing that blood glucose control alone is not sufficient among people with DM. One of the focus areas of current medical research is understanding the various forms of communication among individuals with diabetes and their family members, partners, friends, and health providers, including the reception of love, care, supervision, motivation, and education [29], and conducting in-depth analyses of the possible associated effects and intervention effects on T2DM; the results may have extensive social benefits as well as research and application prospects. The currently known psychological stress-related mechanisms in terms of T2DM development mainly pertain to the autonomic nervous pathways, neuroendocrine mechanisms, and direct effects on the pancreas. Long-term psychological depression promotes the hypothalamic-pituitary-adrenal axis activity to increase cortisol secretion. The process not only reduces glucose utilization and promotes gluconeogenesis, but also raises blood glucose levels by antagonizing insulin production and inhibiting blood glucose utilization [26]. Tomita et al. [30] showed that depression is a chronic inflammatory response through animal experiments, and changes in inflammatory factor levels can affect the normal metabolism of brain tissue cells. Long-term depression and loss may also lead to pessimism, which could, in turn, lead to lower adherence rates to doctors' instructions, and the destruction of the original nursing pattern is likely to have a negative impact on blood glucose control. Similarly, some patients who require insulin monitoring and metering adjustments may experience insulin performance task interruptions or a lack of implementation due to emotional factors. Recent studies have pointed out [31] that the occurrence of depressive symptoms is also closely related to the increase of serum inflammatory factors. It can be speculated that DM has a common mechanism of action with depression. By inhibiting the level of serum inflammatory factors, it can not only effectively improve pathological changes caused by T2DM, but also effectively improve depressive symptoms. Some scholars have suggested that there may be a third factor in the mechanism of the interaction between T2DM and psychological stress. Further research should focus on whether the third factor can produce the same results as psychological depression [32].

## 5. Alcohol Intake

Alcohol intake has become a way of life, with people consuming alcohol to alleviate social pressure. Previous studies have shown a positive correlation between alcohol intake and T2DM development [33], with high intake levels having the potential to damage the liver. The liver is a crucial organ that regulates glucose homeostasis through glycogen synthesis and catabolism. Liver injury can lead to glucose metabolism disorders, resulting in impaired glucose tolerance and DM development. Pathological and ultrastructural abnormalities in chronic alcoholic steatohepatitis are associated with sustained hepatic insulin resistance and proinflammatory cytokine activation [34]. Ethanol intake reduces the sensitivity of islet cells through interference with muscarinic signaling and insulin signaling, resulting in a decrease in the rate of basal insulin secretion. It can be concluded that high alcohol intake levels are closely related to T2DM occurrence. However, another study showed that ethanol intake promotes the expression of insulin signaling and selectively upregulates the insulin transduction pathway by upregulating the expression of intracellular phosphorylated protein kinase B, phosphatidylinositol kinase, and transcription factor pFOX01 and by downregulating the phosphorylation of insulin receptor protein 1 and insulin receptor substrate protein 2 [35]. Moderate drinking can reduce the risk of T2DM. The mechanism is as follows: while moderate drinking can increase the level of high-density lipoprotein, it can lead to significantly reduced levels of serum C-reactive protein, neutrophils, and neutrophil CD64, which may have an anti-inflammatory effect (it is widely accepted that DM is a chronic low-grade inflammatory disease). In addition, moderate drinking can increase the sensitivity of insulin, and ethanol intake can selectively upregulate the insulin signaling pathway in the case of normal blood glucose levels. Therefore, there is a certain dose-effect relationship between alcohol intake and DM development. A cohort study in Korea showed that mild (intake < 5 g/day) and moderate (5 ≤ intake < 30 g/day) alcohol intake reduced the risk of T2DM in men, while high (intake ≥ 30 g/day) alcohol consumption led to increases in the risk of T2DM among men. Therefore, it can be concluded that there is a “J”-type relationship between alcohol consumption and T2DM risk among men [36]. A follow-up survey conducted by Cullmann et al. [37] also showed that heavy drinking increased the risk of pre-diabetes, while the risk of pre-diabetes was lower in those with low or moderate alcohol consumption levels. In women, the risk of DM and its complications is not alleviated by moderate alcohol consumption [38, 39].

## 6. Lack of Exercise

Rapid economic and technological developments have greatly changed the way people commute, resulting in a significant decline in the levels of daily physical activity. Simultaneously, people also do not have the time for regular exercise owing to the accelerated pace of life or are not interested due to various social pressures. It has been concluded that a lack of exercise is among the risk factors for T2DM, and exercise-based intervention is indispensable for diabetic patients. A meta-analysis performed in the United Kingdom of 1,261,991 people enrolled in 28 cohort studies showed that the risk of T2DM associated with moderate-intensity exercise was reduced by 26% with participation in exercise for 150 min per week, by 36% with participation in exercise for 300 min per week, and by 53% with participation in exercise for 800 min per week compared with that in individuals who did not exercise [40]. The American Diabetes Association and the American Movement recommend that diabetic patients participate in at least 150 min of moderate-to-high-intensity exercise per week [41]. Exercise can accelerate the metabolism of glucose and energy, lead to the consumption of a large amount of glycogen, increase the proportion of capillary and muscle fibers, and promote the intake of glucose in the blood. On the completion of an exercise session, blood glucose is stored in the form of glycogen, leading to further blood glucose reduction. Consumption of glycogen reduces the secretion of insulin, promotes the corresponding receptor binding of insulin in the blood circulation to improve insulin resistance, and enhances glucose metabolism. Exercise can also alleviate tension, improve social adaptability, and alter bad lifestyle-related behavior, which can be crucial for the recovery of people with diabetes. The intensity, duration, and volume of exercise should be gradually increased according to a patient's personal situation, and the load should not exceed the patient's ability to withstand it. For patients with DM, daily training should be adhered to. Exercise can be performed across two or three sessions daily (planned such that they do not coincide with the one or two hours following meal intake) and should combine aerobic and anti-resistance movements. Tai Chi, Baduanjin, Five Animal Qigong, and other traditional Chinese exercises have the characteristics of low-intensity and long-term aerobic exercise. These exercise forms are safe and associated with improved health. By coordinating and adjusting limb movements, one can harmonize body parts to echo his/her physiological status, therefore relaxing muscle tissue and bringing further correction of uncoordinated body posture and spirits. Ultimately, one can react to a status in which mind and body are relaxed and coordinated [42, 43]. In recent years, research studies have reported that Qigong training has certain curative effects in cases of chronic diseases such as T2DM [31]. For instance, the sixth section of Baduanjin, a traditional Chinese Qigong, involves the movement “reaching bilateral hands down to feet to nourish kidneys.” By performing waist flexion and extension, as well as massaging the posterior waist and lower limbs, one's governor meridian and bladder meridian will be exercised and exerted. This facilitates the flow of Qi-blood circulation and can cause sympathetic nerve excitation and stimulation of the hypothalamic-pituitary-adrenal pathway, promote increases in insulin secretion by *β*-cells, and lower blood sugar levels; this exercise form also has the effect of regulating water, electrolyte, and acid-base balance [44]. Tai Chi is a traditional Chinese martial art. A large number of studies have confirmed that participation in Tai Chi improves the levels of blood glucose, glycosylated hemoglobin, cholesterol, and other indicators of T2DM and enhances the mechanism of oxidative damage, regulates the balance of the sympathetic and parasympathetic nervous systems, improves immune function, and reduces psychological pressure [45, 46].

## 7. Sleep-Related Factors

In recent years, people's sleep time has shown a downward trend. The average daily sleep time of Chinese residents is 7.20 h, and the proportion of people with sleep insufficiency is 23.60% [47]. Similarly, the proportion of young Americans who sleep for less than 7 hours/day has risen from 15.6% to 37.1% in the last 40 years [48]. The relationship between sleep and T2DM is a topic of international concern, as a lack of sleep can easily lead to a gradual acceleration in the body's insulin resistance. Katano et al. showed that sleep disorders showed a clear association with DM [49]. A cross-sectional study found that people with sleep deprivation had fasting blood glucose levels that were increased by 23%, fasting insulin levels that were 48% higher, and an insulin resistance index (homeostatic model assessment of insulin resistance) that was 82% higher compared to those with sufficient sleep [50]. Lack of sleep is also associated with various metabolic disorders, as it enhances sympathetic activity, boosts catecholamine levels, and inhibits pancreatic function, thereby reducing insulin secretion. Current studies have shown that increased levels of inflammatory factors and mediators in patients with sleep disorders, including tumor necrosis factor-*α*, IL-6, IL-8, high-sensitivity C-reactive protein, transcription factors, and adhesion factors, may affect human health through low-grade inflammation pathways [51, 52]. Increased levels of inflammatory factors are involved in the promotion of insulin resistance by sleep disorders [53]. Difficulties in sleeping reduce the brain's glucose ingestion and insulin sensitivity, impair glucose tolerance, and induce insulin resistance to some extent. The irritability caused by long-term difficulty in falling asleep affects hypothalamic activity, especially in the hypothalamic-pituitary-adrenal axis that is associated with stress [54]. Glucocorticoids are not only responsible for the destructive action of transmembrane glucose transporter 4 (due to which glucose cannot be transported to the cell surface and utilized by the body) but also directly inhibit insulin secretion by islet *β*-cells. Therefore, elevated cortisol levels caused by sleep problems can also trigger T2DM. Other studies have shown that sleep disorders can lead to metabolic disorders; resistin, leptin, adiponectin, and other cytokines have been shown to mediate insulin resistance, also affecting blood glucose levels [55].

## 8. Summary

In summary, there is a two-way relationship between T2DM and the social and natural environments; changes in these environments may induce or aggravate T2DM and vice versa. Poor lifestyle can lead to increased insulin resistance and promote T2DM development in some risk groups or environments. However, improvements in blood glucose control can be achieved through interventions that do not rely on environmental and social aspects. Although some epidemiological and mechanistic studies have shown that the natural environment, social factors, and personal behavior are related to T2DM, existing data are insufficient, and results of studies are currently inconsistent.

Some limitations of this study must be considered when interpreting the results. First, the current epidemiological investigation is mainly based on T2DM, and there are many patients with type 1 diabetes mellitus (T1DM) and pre-diabetes. Therefore, it is necessary to broaden the scope of the study to T1DM and gestational diabetes mellitus and pre-diabetes. Most recent studies are cross-sectional in design. Thus, the causal implications of environment and behavior factors for T2DM should be carefully considered. Prospective studies are needed in the future to determine the causal relationship between these risk factors and diabetes. A third limitation is that the results of one measurement do not necessarily reflect the long-term exposure of the human body, so multiple follow-up and determination are required. Fourth, some studies are unable to distinguish individuals with T1DM from those with T2DM, nor can they prove that the investigators evaluated all participants during the relevant survey. Given that the proportion of T1DM patients among all diabetic patients in Korea is less than 1%, we suspect that their inclusion would have had little effect in some studies [56]. Finally, age, gender, and lifestyle habits mentioned in the experimental and epidemiological studies vary widely and may affect the promotion and comparison of the above risk factors with recent study results. For instance, hormonal effects and low statistical power may have contributed to the differences in findings according to sex; thus, the role of endogenous sex hormones in the development of T2DM may have influenced the gender differences noted in this study [57]. Additionally, the insufficient number of females likely resulted in the negative relationship between high-risk female drinkers and the prevalence of T2DM.

Therefore, it is important to perform a more in-depth exploration of the relationship between the aforementioned factors and T2DM. Moreover, there is an urgent need to conduct systematic and deeper research focusing on the pathological mechanism that connects the environment and individual behavior to T2DM. Future clinically relevant and epidemiological studies aimed at achieving comprehensive, optimized interventions can provide a reliable basis for diagnosis and treatment among clinicians.
